# Global Trends in Application of Stereology as a Quantitative Tool in Biomedical Research

**DOI:** 10.1155/2018/1825697

**Published:** 2018-09-13

**Authors:** Maulilio J. Kipanyula, Alfred S. Sife

**Affiliations:** ^1^Department of Anatomy and Pathology, College of Veterinary Medicine and Biomedical Sciences, Sokoine University of Agriculture, P.O. Box 3016, Chuo Kikuu, Morogoro, Tanzania; ^2^Sokoine National Agricultural Library, Sokoine University of Agriculture, P.O. Box 3022, Chuo Kikuu, Morogoro, Tanzania

## Abstract

Stereology is a quantitative and comparative method that utilizes planes, lines, and points for the estimation of three-dimensional parameters in morphological studies. It primarily focuses on geometrical features of objects such as number, density, length, area, and volume. A scientometric study was conducted to analyze global research trends in application of stereology in biomedical research. Stereology has gained wide application resulting into design-based stereological methods. Data for this study were retrieved from the SCOPUS database. At least 5,732 publications employing stereology as analytical tool were produced in a period of 50 years between 1966 and 2016. Half (2,858; 49.87%) of these publications were produced in the last 12 years from 2005 to 2016. The relative growth rate (RGR) of publications decreased from 1967 (0.69) to 2016 (0.03) whereas the doubling time (DT) increased from 1.00 to 20.56 in the same period. A great majority (5,332; 93.02%) of the publications retrieved from SCOPUS were journal articles in various biomedical fields. The* Journal of Microscopy* tops the list of journals with at least 205 articles. The most productive country was USA with at least 1663 (23.10%) publications and Aarhus Universitet tops the list of institutions with at least 306 publications. J.R. Nyengaard was the most prolific author who contributed at least 125 publications. The highly cited article had a total of 2,054 citations with an average of over 82 citations per year. Given the growing importance of stereology in biomedical research, it is necessary to promote its application among scholars.

## 1. Introduction

Quantitative investigation of images taken from light microscopy observation is one of the pillars of biological and biomedical investigation and is achieved through stereological methods. Stereology refers to three-dimensional interpretation of two-dimensional cross sections of materials or tissues. Primarily these are practical techniques for extracting quantitative information about a three-dimensional material from measurements made on two-dimensional planar sections of the material. Certainly, the application of stereological techniques provides a pragmatic approach in histological analyses and has driven most of advances in morphometric and biological image analyses. It is a quantitative and comparative method that utilizes planes, lines, and points for the estimation of three-dimensional parameters in morphological studies. It primarily focuses on geometrical features of objects such as number, density, length, area, and volume [1, 2]. The technique derives three-dimensional features of an object or matter ordinarily observed as a profile in two dimensions. The use of two-dimensional profiles in morphometric studies is prone to errors as it does not directly reflect the object numbers and size. Consequently, significant error can be made during interpretation of the quantitative data obtained from profiles. Previous studies have shown that when various objects of one, two, or three dimensions are subjected to a two-dimensional section plane, the resulting profiles are one dimension less than the original. Certainly, a two-dimension surface produces a one-dimension linear profile while a three-dimension object gives rise to two-dimension planar profiles [2, 3]. Therefore, application of stereology allows researchers to effectively and efficiently gather accurate and unbiased quantitative data. Furthermore, stereological methods produce numerical and reproducible results to allow comparison among different experimental groups under different treatments. Strict sampling strategy, isotropy, and randomness in stereology minimize errors and biasness commonly encountered in quantitative analyses involving profiles [4–6].

Indeed, stereological estimations are the gold standard for quantitative studies of volume, length, and surface area as dimensional parameters in biomedical experiments. Over the years, stereology has gained wide application resulting into design-based stereological methods. These are among recent advances which have allowed generation of high quality quantitative estimates of cell and tissue elements from light microscopy images [7, 8]. Design-based stereology involves rigorous quantitative analysis of the size, shape, and number of objects. Its application permits validation and rejection of experimental hypotheses in biomedical research. The most important strength of this approach is that it produces results that are unbiased, efficient, and more reliable. Unbiased stereology guarantees consistency and dependability of quantitative analytical results produced in the laboratory and reported in scientific publications. It is one of important analytical tools for predicting functional and structural alterations in health, and unhealthy tissues and organs through unbiased mathematical descriptions of their relationships.

Cell counting is one of the key activities in stereological analyses and is time-consuming. However, recent advances have resulted into automation of some of these analyses. For example, the proportionator is a new stereological sampling method combining automatic image analysis and nonuniform sampling procedures. In this case the autodisector on virtual slides combines automatic generation of dissector pairs with the use of digital images [9]. This results into accurate and reproducible data.

The present study employed scientometric techniques to analyze global trends on the application of stereology in biomedical research. Scientometric methods are usually used to evaluate research performance in terms of publications productivity and offer a comprehensive assessment of scientific research trends of any topic. These methods enable quantitative analysis of the research output of individuals, institutions, or countries [18, 19]. Disciplines' progress and reputation can also be tracked based on research performance [20, 21]. In the present study, emphasis was given on the growth of publications over time and their distribution by source, type, country of origin, and authors. This study therefore provides an understanding of the status and trends on the application of stereology as a key tool in biomedical research. Specifically, the study examined growth pattern of research output; distribution of publications by type and subjects; country-wise and institution-wise distribution of publications; most prolific authors; and highly cited papers. The present study findings depict worldwide trends and progress on the application of stereological techniques over the years. This forms a basis for decisions related to the promotion of stereological methods as advances for quantitative estimates of cell and tissue elements. The findings can also inform decisions on research policies, priority research areas, training needs (especially where there is less application of stereology), and resource allocations in this discipline.

## 2. Materials and Methods

Data for this study were retrieved from the SCOPUS international and multidisciplinary indexing database on 29^th^ December 2016. SCOPUS was chosen because it is a relatively large database with a more expanded spectrum of journals compared with other databases such as PubMed and Web of Science. Only scholarly publications in English, namely, journal articles, reviews, conference papers, book chapters, and books, were retrieved. A search query was constructed and employed to retrieve data that contained the term* stereology* and searching was limited to the document title, abstract, and keywords. These three sections—title, abstract, and keywords—always capture the essence of a publication and reflect the content of the main text. In other words, any paper on stereology is expected to have the term* stereology* in one or all these three sections. In addition, most search engines, databases, and journal websites (including SCOPUS) use the words found in the title, abstract, and keyword sections to enable readers retrieve papers. The search string was also refined by “subject area” by limiting it to various biomedical sciences available in SCOPUS database including medicine, biochemistry, neurology, pharmacy, immunity, veterinary, dentistry, and nursing.

The following strings were used to retrieve data on stereology from SCOPUS database: (TITLE-ABS-KEY(stereology) AND (LIMIT-TO (SUBJAREA, ＇＇MEDI＇＇) OR LIMIT-TO (SUBJAREA, ＇＇BIOC＇＇) OR LIMIT-TO (SUBJAREA, ＇＇NEUR＇＇) OR LIMIT-TO (SUBJAREA,＇＇PHAR＇＇) OR LIMIT-TO (SUBJAREA, ＇＇IMMU＇＇) OR LIMIT-TO (SUBJAREA, ＇＇HEAL＇＇) OR LIMIT-TO (SUBJAREA, ＇＇VETE＇＇) OR LIMIT-TO (SUBJAREA, ＇＇DENT＇＇) OR LIMIT-TO (SUBJAREA, ＇＇NURS＇＇)) AND (LIMIT-TO (DOCTYPE, ＇＇ar＇＇) OR LIMIT-TO (DOCTYPE, ＇＇re＇＇) OR LIMIT-TO(DOCTYPE, ＇＇cp＇＇) OR LIMIT-TO(DOCTYPE, ＇＇ch＇＇)) AND (LIMIT-TO (LANGUAGE, ＇＇English＇＇)) AND (LIMIT-TO(SRCTYPE, ＇＇j＇＇) OR LIMIT-TO (SRCTYPE, ＇＇k＇＇) OR LIMIT-TO (SRCTYPE, ＇＇b＇＇) OR LIMIT-TO (SRCTYPE, ＇＇p＇＇))). The collected data were compiled using MS Excel and statistical analysis such as frequency and percentage distribution and scientometric techniques such as relative growth rate (RGR) and doubling time (dt) were computed.

## 3. Results and Discussions

### 3.1. Growth of Publications

**Figure 1:**
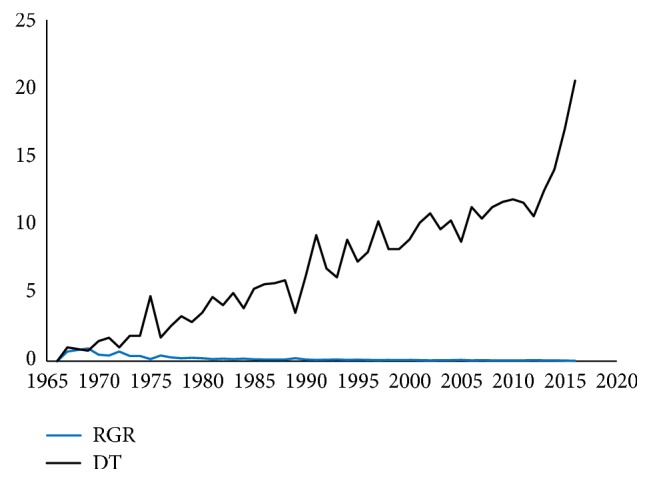
Relative growth rate and doubling time for research output. The RGR is shown in blue and the DT in black.

The findings show that at least 5,732 publications were retrieved worldwide, giving an average of 115 publications per year (Table 1). The oldest publication was produced in 1966 and publications were retrieved for each year with exception of the year 1968. Half (2,858; 49.87%) of the publications were produced in the last 12 years from 2005 to 2016 and the year 2012 had the highest number of publications (303; 5.29%) as shown in Table 1. The growth of publications was also analyzed based on two scientometric parameters, namely, the relative growth rate (RGR) and doubling time (DT). RGR is the increase in the number of publications per unit of time and it is calculated using the formula RGR = (ln⁡N_2_ - ln⁡N_1_)/ (t_2_ –t_1_), where N_2_ and N_1_ are the cumulative number of publications in the years t_2_ and t_1_. The parameter doubling time (DT) indicates the time required for publications to become double of the existing amount. DT is related to RGR in that if the number of articles doubles then the difference between the logarithms of numbers at the beginning and end of that period is 693 and it is calculated as DT = 0.693/RGR [21]. It is observed from Table 1 and Figure 1 that RGR has shown a slightly downward trend from 1967 (0.69) to 2016 (0.03) whereas DT had an increased trend from 1.00 to 20.56 in the same period. This means that although the number of publications increased since 1966, its rate of growth slightly decreased while the corresponding doubling time increased.

### 3.2. Publications Types

**Figure 2:**
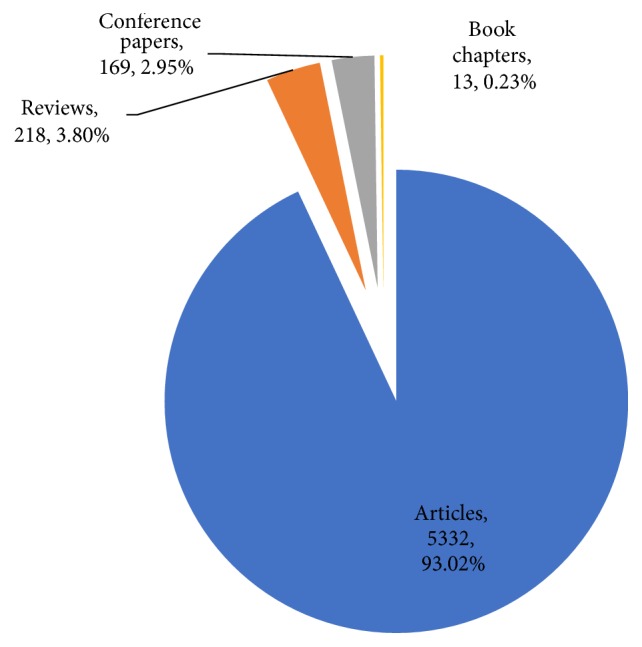
Relative growth rate and doubling time for research output. The RGR is shown in blue and the DT in black.

A great majority (5,332; 93.02%) of the publications retrieved from SCOPUS were journal articles (Figure 2). This is expected because peer reviewed journals are the major communication channels for original scientific research findings. This confirms the fact that, as a research technique, stereology was mostly employed in research whose results are often published as journal articles.

### 3.3. Subject Areas

The subject-wise breakup of publications based on subject categories in SCOPUS shows that most publications were in biomedical sciences such as medicine (38.62%), biochemistry, genetics and molecular biology (20.74%), neuroscience (19.42%), pharmacology, toxicology and pharmaceutics (3.40%), immunology and microbiology (1.52%), health (1.14%), veterinary (0.87%), dentistry (0.52%), and nursing (0.31%). The findings also show that the application of stereology is not confined to biomedical sciences only. Other disciplines such agricultural sciences (6.18%) have also applied stereology (Table 2).

### 3.4. Core Journals

Of the total world output in research that applied stereology as a quantitative tool, 62.69% (3,599) publications appeared as articles in 160 peer reviewed journals. These 160 journals had 7 or more research articles each. The top 25 journals collectively produced about one-third (1,770; 31%) of the journal articles and they accounted for 32 to 205 articles each. The* Journal of Microscopy* tops the list with 205 articles, followed by the* Journal of Comparative Neurology* (192 articles) and the* Brain Research* (134 articles) (Table 3). According to Bradford's Law of Scattering, there are core journals in every discipline that are frequently referred to by researchers because they always contain relevant articles in the respective discipline. Bradford's Law states that if scientific journals are arranged in order of their decreasing productivity of articles on a given subject, they may be divided into a nucleus of periodicals more particularly devoted to the subject, and several “groups” or “zones” containing the same number of articles as the nucleus. The number of periodicals in the nucleus and succeeding zones will be in the ration of 1:n:n^2^, where “n” is a multiplier. Bradford multiplier is the ratio of the number of periodical titles in any group to the number of periodical titles in any immediately preceding group [22, 23]. In this study, the total numbers of journal articles were divided into three equal zones in which 12 journals covered 1,218 articles, next 41 journals covered 1,210 articles, and the next 107 journals covered 1,171 articles. That means the ratio of journals in each zone was 12:41:107 or 1:3.4:8.9 (Table 4) and the mean value of Bradford's multiplier computed was 3. The computed expected ratio in three successive zones was 1:3:9 which indicates adherence to Bradford's law. This suggests that although there are many journals that publish research that applies stereology, there are a few core journals that are mostly preferred by researchers.

### 3.5. Country-Wise Research Output

**Figure 3:**
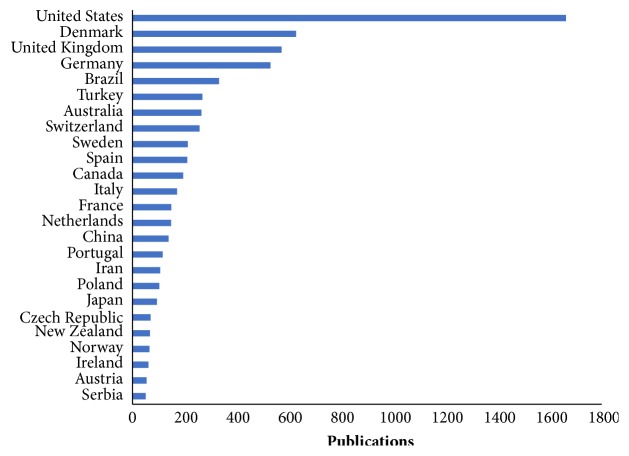
Distribution of publications by country. The bar charts represent the number of publications per country.

The study findings indicate that more than 85 countries have produced research in which stereology was applied as a quantitative technique.  Figure 3 shows the distribution of research output for the top 25 countries based on the “normal counting method” whereby, in case of multiple authors from different countries, each author receives a full count for joint publications. These top 25 countries have produced an overwhelming majority (90.58%) of the publications, contributing between 51 and 1,663 publications each during the 1966–2016 period. United States of America (USA) is the most productive country on research that applies stereology as quantitative tool, with at least 1663 (23.10%) publications followed by Denmark with 627 (8.71%) and United Kingdom (UK) with 572 (7.95%). Brazil (332; 4.61%) is the only country from the developing world which is in the top five. Only a few African countries had some stereology-related publications with South Africa topping the list with only 24 publications. These findings indicate that the research that applies stereology as a quantitative technique is not scattered to many countries particularly in Africa. Low level of utilization of stereology may have negative implication with respect to quality of publications in morphometrical studies. This trend calls for concerted efforts to promote stereology in the developing world.

### 3.6. Most Productive Authors

**Figure 4:**
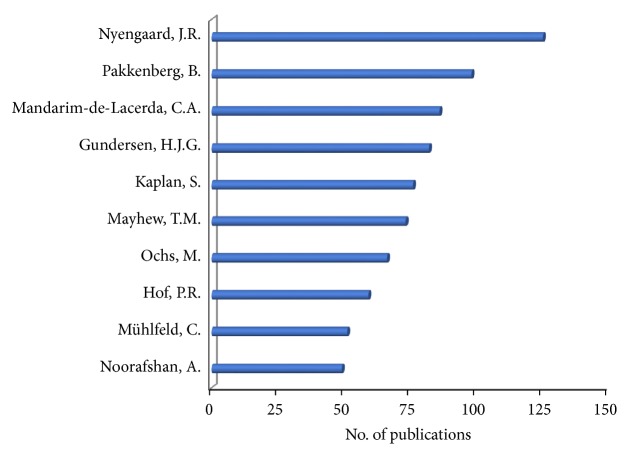
Distribution of publications by author. The bar charts represent the number of publications per author.

Figure 4 shows the most prolific contributors of the research output in which stereology was applied as a quantitative technique. J.R. Nyengaard has contributed 125 publications, followed by B. Pakkenberg with 98 publications and C.A. Mandarim-de-Lacerda with 86 publications. The top 10 scholars have collectively produced at least 765 publications, each contributing between 51 and 125 publications. These are world reknown research groups in the field of stereology.

### 3.7. Institutions-Wise Research Output

**Figure 5:**
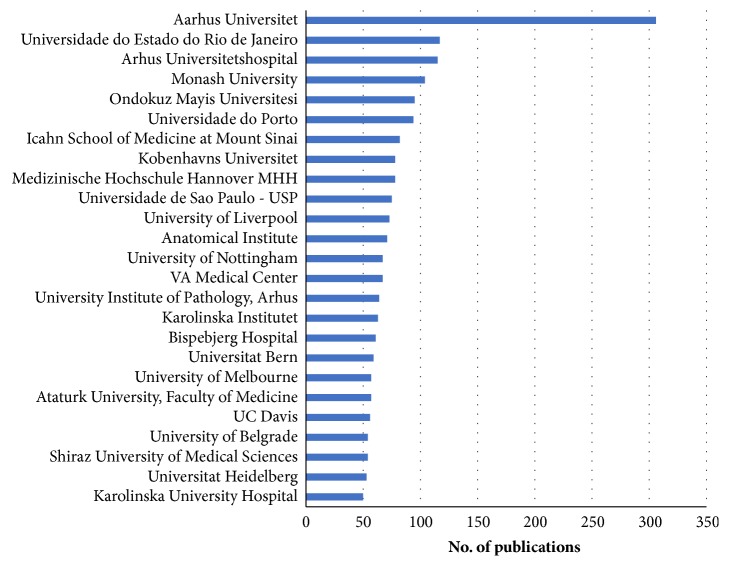
Distribution of publications by institution affiliation. The bar charts represent the number of publications per university/institution.

The identification of prolific institutions is also an important aspect of scientometric studies. The number of prolific research institutions from a country often depends upon factors such as grants received from the government, industry support for research in a particular area, economic growth of a country, or interests of the scientists working in the field. In the present study, the total output came from 158 institutions located in different parts of the world.  Figure 5 indicates the 25 most prolific institutions that contributed 50 or more publications. Aarhus Universitet tops the list of institutions with at least 306 publications followed by Universidade do Estado do Rio de Janeiro (117 publications) and Arhus Universitets hospital (115 publications). These 25 institutions produced over one-third (35.76%; 2,050) of the total global output. Although USA ranked number one among countries with large number of publications, only one institution (UC Davis) was in the top 25 universities.

### 3.8. Citation Counts

Citation analysis measures the impact of each publication by counting the number of times they are cited by other articles. High levels of citation to a scientific publication are interpreted as signs of scientific influence, impact, and visibility. The top 10 highly cited publications had citations ranging from 844 to 2,054. Of the 10 highly cited papers, 8 were published as journal articles, one was conference article, and another one was a technical report (Table 5). Of the 8 highly cited journal articles, three were published in the* Journal of Neuroscience*. The most highly cited article was titled “Unbiased Stereological Estimation of the Total Number of Neurons in the Subdivisions of the Rat Hippocampus Using the Optical Fractionator” published in 1991 in the* Anatomical Record*. This article had received a total of 2,054 citations with an average of over 82 citations per year.

## 4. Conclusion and Recommendations

This study employed scientometric methods to analyze global trends in the application of stereology as quantitative tool in biomedical research. The number of publications produced in 50 years starting 1966 to 2016 increased from one to at least 5,732 publications. However, the results have also demonstrated a slight decrease in the relative growth rate of publications. Most biomedical publications retrieved from SCOPUS were articles from at least 160 peer reviewed journals. The top 25 journals collectively produced one-third of the journal articles. This trend showed adherence to Bradford's Law which suggests that in every subject there are some journals that are preferred more by scholars. The study findings also show that research activity that applies stereology as quantitative tool is still low in many countries and institutions particularly in Africa. The top 10 most productive scholars have collectively produced 765 publications. Journal articles were the most highly cited publications. Given the growing importance of stereology as a quantitative tool in biomedical research, it is necessary to promote its application among scholars particularly in the developing world. Since stereological methods are gold standard for quantitative studies, knowledge of the basic principles of stereology is required and will consequently increase the number of quantitative studies with reproducible data in biomedical sciences. Low utilization of the tool calls for the need for strategic capacity building initiatives for scientists in the developing world. Furthermore, the findings from this study provide baseline data and form a strong basis for decisions related to the promotion of stereological methods as recent advances for quantitative studies. The findings can also inform decisions on research policies, priority research areas, training needs, and preferential institutional resource allocations in this discipline.

## Figures and Tables

**Table 1:** Publications which applied stereology as quantitative tool during the past 50 years. The number of publications per year, cumulative frequency, relative growth rate (RGR), and doubling time (DT) are presented.

Year	Number of publications	Percent	Cumulative frequency	Relative Growth Rate	Doubling Time
1966	1	0.02	1	-	-
1967	1	0.02	2	0.69	1.00
1969	3	0.05	5	0.92	0.76
1970	3	0.05	8	0.47	1.47
1971	4	0.07	12	0.41	1.71
1972	12	0.21	24	0.69	1.00
1973	11	0.19	35	0.38	1.84
1974	16	0.28	51	0.38	1.84
1975	8	0.14	59	0.15	4.76
1976	29	0.51	88	0.40	1.73
1977	27	0.47	115	0.27	2.59
1978	27	0.47	142	0.21	3.29
1979	39	0.68	181	0.24	2.86
1980	39	0.68	220	0.20	3.55
1981	35	0.61	255	0.15	4.69
1982	47	0.82	302	0.17	4.10
1983	45	0.79	347	0.14	4.99
1984	68	1.19	415	0.18	3.87
1985	58	1.01	473	0.13	5.30
1986	62	1.08	535	0.12	5.63
1987	69	1.20	604	0.12	5.71
1988	75	1.31	679	0.12	5.92
1989	147	2.56	826	0.20	3.54
1990	97	1.69	923	0.11	6.24
1991	72	1.26	995	0.08	9.23
1992	107	1.87	1102	0.10	6.78
1993	132	2.30	1234	0.11	6.13
1994	100	1.74	1334	0.08	8.89
1995	133	2.32	1467	0.10	7.29
1996	133	2.32	1600	0.09	7.99
1997	112	1.95	1712	0.07	10.24
1998	151	2.63	1863	0.08	8.20
1999	164	2.86	2027	0.08	8.21
2000	164	2.86	2191	0.08	8.91
2001	155	2.70	2346	0.07	10.14
2002	155	2.70	2501	0.06	10.83
2003	186	3.24	2687	0.07	9.66
2004	187	3.26	2874	0.07	10.30
2005	237	4.13	3111	0.08	8.75
2006	197	3.44	3308	0.06	11.29
2007	227	3.96	3535	0.07	10.44
2008	224	3.91	3759	0.06	11.28
2009	230	4.01	3989	0.06	11.67
2010	240	4.19	4229	0.06	11.86
2011	260	4.54	4489	0.06	11.61
2012	303	5.29	4792	0.07	10.61
2013	273	4.76	5065	0.06	12.51
2014	256	4.47	5321	0.05	14.05
2015	221	3.86	5542	0.04	17.03
2016	190	3.31	5732	0.03	23.10

**Table 2:** Distribution of publications by subject area. The number of publications and percentage distribution are presented.

Subject area	Number of publications	Percent
Medicine	3650	38.62
Biochemistry, Genetics and Molecular Biology	1960	20.74
Neuroscience	1836	19.42
Agricultural and Biological Sciences	584	6.18
Pharmacology, Toxicology and Pharmaceutics	321	3.40
Immunology and Microbiology	144	1.52
Physics and Astronomy	133	1.41
Health Professions	108	1.14
Psychology	107	1.13
Environmental Science	93	0.98
Veterinary Sciences	82	0.87
Materials Science	69	0.73
Mathematics	54	0.57
Computer Science	52	0.55
Dentistry	49	0.52
Engineering	39	0.41
Decision Sciences	38	0.40
Chemical Engineering	32	0.34
Nursing	29	0.31
Social Sciences	22	0.23
Chemistry	17	0.18
Arts and Humanities	14	0.15
Earth and Planetary Sciences	8	0.08
Multidisciplinary	7	0.07
Energy	4	0.04

Total	*∗*9,452	100.00

*∗*The number of publications exceeds 5,732 because some publications cover more than one subject category.

**Table 3:** Distribution of publications by source. The name of the journal and number of publications contributed are shown.

S. No.	Journal title	Number of publications
1	Journal of Microscopy	205
2	Journal of Comparative Neurology	192
3	Brain Research	134
4	Neuroscience	114
5	Anatomical Record	89
6	Journal of Neuroscience	82
7	Cell and Tissue Research	79
8	Journal of Anatomy	72
9	Plos One	68
10	Neurobiology of Aging	63
11	Acta Stereologica	61
12	Journal of Neuroscience Methods	59
13	Experimental Neurology	58
14	Acta Pathologica, Microbiologica, et Immunologica Scandinavica	57
15	Bulletin of Experimental Biology and Medicine	51
16	Analytical and Quantitative Cytology and Histology	50
17	European Journal of Neuroscience	45
18	Neuroscience Letters	43
19	Placenta	39
20	Neurobiology of Disease	38
21	Image Analysis and Stereology	36
22	Acta Neuropathologica	35
23	Annals of Anatomy	34
24	Microscopy Research and Technique	34
25	American Journal of Physiology Lung Cellular and Molecular Physiology	32

	Total	1,770

**Table 4:** Bradford distribution. The zones, number of articles, and number of journals are summarized.

Zone	Number of articles	Number of journals
I	1218	12
II	1210	41
III	1171	107

Total	83	38

**Table 5:** Highly cited publications. The number of citations and average citation per year are presented.

S. No.	Publication	Number of citations	Citations per year
1	[10] West, M.J., Slomianka, L., Gundersen, H.J.G. (1991). Unbiased stereological estimation of the total number of neurons in the subdivisions of the rat hippocampus using the optical fractionator. *The Anatomical Record*, 231 (4), pp. 482-497.	2054	82.16
2	[5] Gundersen, H.J.G., Bendtsen, T.F., Korbo, L., Marcussen, N., Moller, A., Nielsen, K., Nyengaard, J.R., Pakkenberg, B., Sorensen, F.B., Vesterby, A., West, M.J. (1988). Some new, simple and efficient stereological methods and their use in pathological research and diagnosis.* APMIS*, 96 (5), pp. 379-394.	2186	78.07
3	[11] Sheline, Y.I., Wang, P.W., Gado, M.H., Csernansky, J.G., Vannier, M.W. (1996). Hippocampal atrophy in recurrent major depression. *Proceedings of the National Academy of Sciences of the United States of America*, 93 (9), pp. 3908-3913.	1351	67.55
4	[12] Gundersen, H.J.G., Bagger, P., Bendtsen, T.F., Evans, S.M., Korbo, L., Marcussen, N., Moller, A., Nielsen, K., Nyengaard, J.R., Pakkenberg, B., Sorensen, F.B., Vesterby, A., West, M.J. (1988). The new stereological tools: Disector, fractionator, mucleator and point sampled intercepts and their use in pathological research and diagnosis. *APMIS*, 96 (10), pp. 857-881.	1767	63.11
5	[13] Rouquerol, J., Avnir, D., Fairbridge, C.W., Everett, D.H., Haynes, J.M., Pernicone, N., Ramsay, J.D.F., Sing, K.S.W. and Unger, K.K. (1994). Recommendations for the characterization of porous solids (Technical Report). *Pure and Applied Chemistry*, 66(8), pp.1739-1758.	1341	60.95
6	[14] Sheline, Y.I., Sanghavi, M., Mintun, M.A., Gado, M.H. (1999). Depression duration but not age predicts hippocampal volume loss in medically healthy women with recurrent major depression. *Journal of Neuroscience*, 19 (12), pp. 5034-5043	1002	58.94
7	[15] Sterio, D.C. (1984). The unbiased estimation of number and sizes of arbitrary particles using the disector. *Journal of Microscopy*, 134 (2), pp. 127-136.	1885	55.44
8	[16] Gómez-Isla, T., Price, J.L., McKeel Jr., D.W., Morris, J.C., Growdon, J.H., Hyman, B.T. (1996). Profound loss of layer II entorhinal cortex neurons occurs in very mild Alzheimer's disease. *Journal of Neuroscience*, 16 (14), pp. 4491-4500.	1001	50.05
9	[17] Kempermann, G., Kuhn, H.G., Gage, F.H. (1998). Experience-induced neurogenesis in the senescent dentate gyrus. *Journal of Neuroscience*, 18 (9), pp. 3206-3212.	767	42.61
10	Coggeshall, R.E., Lekan, H.A. (1996). Methods for determining numbers of cells and synapses: A case for more uniform standards of review. *Journal of Comparative Neurology*, 364 (1), pp. 6-15.	844	42.20

## Data Availability

The data used to support the findings of this study are included within the article.

## Additional Points

*Limitations of the Study.* The most important limitation of this study lies in the fact that publications were retrieved from the SCOPUS database alone. This means that publications indexed in other databases were not captured. Also, only papers in English that had the term stereology in the title, abstract, and keyword sections were retrieved. Nevertheless, the study gives a clear picture about the global trends of research that applies stereology as a quantitative tool.

## References

[1] Baddeley A., Jensen E. B. (2004). *Boca Raton*.

[2] Altunkaynak B. Z., Önger M. E., Altunkaynak M. E., Ayranci E., Canan S. (2012). A brief introduction to stereology and sampling strategies: Basic concepts of stereology. *NeuroQuantology*.

[3] Howard V., Reed M. (2004). Unbiased Stereology: Three-Dimensional Measurement in Microscopy. *Advanced Methods*.

[4] Gundersen H. J. G., Jensen E. B. V., Kiêu K., Nielsen J. (1999). The efficiency of systematic sampling in stereology-reconsidered. *Journal of Microscopy*.

[5] Gundersen H. J. G., Bendtsen T. F., Korbo L. (1988). Some new, simple and efficient stereological methods and their use in pathological research and diagnosis. *APMIS-Acta Pathologica, Microbiologica et Immunologica Scandinavica*.

[6] Mandarim-de-Lacerda C. A. (2003). Stereological tools in biomedical research. *Anais da Academia brasileira de Ciências*.

[7] Kaplan S., Canan S., Aslan H., Ünal B., Sahin B. (2001). A simple technique to measure the movements of the microscope stage along the x and y axes for stereological methods. *Journal of Microscopy*.

[8] Geuna S., Herrera-Rincon C. (2015). Update on stereology for light microscopy. *Cell and Tissue Research*.

[9] Keller K. K., Andersen I. T., Andersen J. B. (2013). Improving efficiency in stereology: A study applying the proportionator and the autodisector on virtual slides. *Journal of Microscopy*.

[10] West M. J., Slomianka L., Gundersen H. J. G. (1991). Unbiased stereological estimation of the total number of neurons in the subdivisions of the rat hippocampus using the optical fractionator. *Anatomical Record*.

[11] Sheline Y. I., Wang P. W., Gado M. H., Csernansky J. G., Vannier M. W. (1996). Hippocampal atrophy in recurrent major depression. *Proceedings of the National Acadamy of Sciences of the United States of America*.

[12] Gundersen H. J. G., Bagger P., Bendtsen T. F. (1988). The new stereological tools: disector, fractionator, mucleator and point sampled intercepts and their use in pathological research and diagnosis. *APMIS-Acta Pathologica, Microbiologica et Immunologica Scandinavica*.

[13] Rouquerol J., Avnir D., Fairbridge C. (1994). Recommendations for the characterization of porous solids (Technical Report). *Pure and Applied Chemistry*.

[14] Sheline Y. I., Sanghavi M., Mintun M. A., Gado M. H. (1999). Depression duration but not age predicts hippocampal volume loss in medically healthy women with recurrent major depression. *Journal of Neuroscience*.

[15] Sterio D. C. (1984). The unbiased estimation of number and sizes of arbitrary particles using the disector. *Journal of Microscopy*.

[16] Gómez-Isla T., Price J. L., McKeel D. W., Morris J. C., Growdon J. H., Hyman B. T. (1996). Profound loss of layer II entorhinal cortex neurons occurs in very mild Alzheimer's disease. *The Journal of Neuroscience*.

[17] Kempermann G., Kuhn H. G., Gage F. H. (1998). Experience-induced neurogenesis in the senescent dentate gyrus. *The Journal of Neuroscience*.

[18] Zyoud S. H., Al-Jabi S. W., Sweileh W. M. (2015). Scientific publications from Arab world in leading journals of Integrative and Complementary Medicine: a bibliometric analysis. *BMC Complementary and Alternative Medicine*.

[19] López-muñoz F., Srinivasan V., Gutiérrez-soriano A., Shen W. W., Rubio G., Álamo C. (2016). A Bibliometric Analysis of Scientific Research on Atypical Antipsychotic Drugs in India during 1998-2013. *Molecules and Medicinal Chemistry*.

[20] Ingram R., Petersen R. (1991). The Accounting Profession and the Market for Accounting Teachers. *Accounting Educators' Journal*.

[21] Read W., Rama D., Raghunandan K. (1998). Are Publication Requirements for Accounting Faculty Promotions Still Increasing?. *Issues in Accounting Education*.

[22] Vickery B. C. (2015). Bradfords Law of Scattering. *Journal of Documentation*.

[23] Mahapatra G. (1994). Correlation Between Growth of Publications and Citations: A Study Based on Growth Curves. *Annals of Library science and Documentation*.

